# Multi-Omics and Artificial Intelligence in Cardiovascular Medicine: From Mechanistic Insights to Clinical Translation

**DOI:** 10.3390/biomedicines14061301

**Published:** 2026-06-08

**Authors:** Ewelina Młynarska, Kinga Bojdo, Oliwia Mazur, Kacper Pawlak, Aleksandra Przybylak, Natalia Kustosik, Katarzyna Krawiranda, Jacek Rysz, Beata Franczyk

**Affiliations:** 1 Department of Nephrocardiology, Medical University of Lodz, 90-419 Lodz, Poland; 2 Department of Nephrology, Hypertension and Internal Medicine, Medical University of Lodz, 90-549 Lodz, Poland

**Keywords:** cardiovascular disease, multi-omics, artificial intelligence, biomarkers, clinical translation, non-coding RNA, molecular patophysiology

## Abstract

**Background:** Cardiovascular diseases (CVDs) remain the leading global cause of mortality, yet a critical “translational gap” persists: Conventional biomarkers often fail to detect subclinical stages or predict individual disease trajectories. While single-omics studies have proliferated, the field lacks a unified framework synthesizing these molecular layers with advanced computational intelligence. **Aim:** This review addresses this gap by evaluating the synergistic integration of multi-omics and Artificial Intelligence (AI) to transition from descriptive markers toward predictive precision cardiology. **Scope:** Evidence from non-coding RNA networks (miRNAs, lncRNAs) and exosomal trafficking is synthesized alongside a critical assessment of Machine Learning (ML) architectures, including supervised, unsupervised, and deep learning (DL) models. **Findings:** Unlike traditional reviews, this work delineates the specific pipelines required to deconvolute high-dimensional signatures—such as TMAO, acylcarnitines, and cardiac-enriched miRNAs—into actionable risk models for heart failure (HF) and post-infarction outcomes. The primary barrier to clinical translation is identified not as data scarcity but as the lack of standardized bioinformatic workflows and model interpretability. **Conclusions:** This review distinguishes itself by proposing an integrated molecular–computational framework that prioritizes Explainable AI (XAI) and standardized multi-omic protocols. Such a shift is essential to bridge the gap between high-dimensional biological insights and routine clinical decision-making.

## 1. Introduction

### 1.1. Global Burden and Complexity of Cardiovascular Diseases

CVDs are the preeminent cause of global mortality and a principal contributor to disability worldwide. The burden associated with CVDs has exhibited a consistent and substantial increase across most nations since 1990. This upward trajectory is fundamentally driven by cumulative demographic pressures, notably population growth and aging, as well as heightened exposure to detrimental risk factors.

The comprehensive scale and drivers of the CVD burden from 1990 to 2023 were meticulously quantified by the multinational Global Burden of Disease (GBD) 2023 study. Employing available data and robust statistical models, this collaborative effort provided population-level burden estimates for 204 countries and territories.

The GBD 2023 reaffirmed CVD as the leading cause of global disease burden and death, with a disproportionately high incidence observed in regions categorized as having low, low-middle, and middle Socio-demographic Index (SDI). Significant inter-country variability in this burden persists, even among regions sharing similar developmental status. This disparity is substantially attributable to inadequate control of known, modifiable risk factors, led predominantly by metabolic risks [1]. Successfully reducing the burden of CVD to meet global targets necessitates the implementation of effective health systems and public health strategies to mitigate these pervasive risks.

The persistent nature of this global challenge is further highlighted by long-term projections, such as those based on GBD 2019 data, which forecast a substantial 73.4% rise in crude mortality by 2050, resulting in an estimated 35.6 million cardiovascular deaths. This escalation is primarily driven by the aging global populace. While the decrease in age-standardized mortality reflects improvements in post-diagnosis medical care, the relatively constant age-standardized prevalence suggests that current preventative efforts are failing to substantially curb disease incidence. Given that the continued burden is largely attributed to atherosclerotic diseases, a critical translational gap is evident: the existing framework, reliant on conventional diagnostic and prognostic tools, is insufficient to effectively mitigate CVD incidence at the early or subclinical stages [2].

This clinical inadequacy necessitates the exploration of more sensitive, mechanistically relevant biomarkers.

### 1.2. Limitations of Conventional Biomarkers

Conventional biomarkers have served a foundational role in CVD detection and risk assessment, though their inherent limitations underscore the need for more accurate indicators. For instance, Low-Density Lipoprotein Cholesterol (LDL-C), despite being central to atherosclerosis assessment, exhibits restricted specificity, as disease progression is also driven by factors such as genetics and inflammation. Similarly, High-Sensitivity C-Reactive Protein (hsCRP), a measure of chronic inflammation, lacks specificity because its levels can be elevated by various non-cardiac conditions, hindering accurate individual risk prediction. Critically, these conventional markers often become abnormal only after significant disease progression has occurred, which impedes the implementation of timely, preventative interventions. Consequently, the shortcomings of traditional biomarkers have driven the search for novel indicators, including genetic markers, microRNAs, metabolomics, and advanced imaging, to enhance the accuracy and predictive value of cardiovascular risk assessment [3]. This identified diagnostic gap is now being rapidly addressed by the omics revolution, which is yielding a diverse array of mechanistically relevant biomarkers. However, the sheer volume and complexity of data generated by multi-omics approaches—required to validate markers like miRNAs and metabolomic signatures—present a significant translational barrier, necessitating the integration of AI and ML to efficiently process and translate these vast datasets into clinical utility [4].

### 1.3. The Omics Revolution: Bridging the Diagnostic Gap

The necessity for highly accurate and mechanistically driven risk assessment has positioned the omics revolution as the future of CVD diagnostics. By moving beyond single-marker approaches, disciplines like genomics, transcriptomics, proteomics, and metabolomics offer an unprecedented ability to capture the complex molecular state of the patient, providing granular insights into pathways like inflammation, fibrosis, and metabolic dysregulation. This integrative molecular profiling allows for the identification of sophisticated signatures, such as microRNAs (miRNAs), long non-coding RNAs (lncRNAs), and specific lipid species, which are proving far more powerful for early disease detection and refining risk stratification than traditional tools. Ultimately, the integration of these multi-omics data streams promises to resolve the current diagnostic gap, enabling truly personalized preventative strategies and advancing precision cardiovascular medicine [5].

By integrating ML algorithms with this vast omics data, clinicians gain the power to project CVD trajectories, anticipate adverse events, and predict patient survival outcomes with greater precision than traditional methods. However, despite the rapid enhancement in analytical efficiency and predictive power offered by ML-based integrative algorithms, several persistent challenges must be addressed to achieve their widespread application in routine clinical practice [6]. These challenges include the crucial requirements for high-quality data sets, suitable models and algorithms, and the creation of standardized methods for data processing and analysis to ensure an accurate perception of complicated biological systems. It is therefore crucial to build collaborative and multidisciplinary research efforts, involving experts in biology, computer science, and statistics, to develop solid, open-source procedures for data analysis and interpretation in order to fully utilize AI and data processing in multi-omics analysis [7].

This article, therefore, provides a comprehensive review of the emerging molecular and computational framework for CVD diagnosis and prognosis, examining the mechanistic basis of novel omics biomarkers, the state-of-the-art applications of AI/ML in biomarker discovery and integration, and the critical steps required for their successful clinical translation.

### 1.4. Sex-Specific Molecular Signatures and AI Bias in Precision Cardiovascular Medicine

Importantly, growing evidence indicates that sex-specific molecular and transcriptomic differences substantially influence cardiovascular disease susceptibility, progression, and therapeutic response. Biological sex modulates several key mechanisms involved in CVD pathophysiology, including activation of the nuclear factor kappa-light-chain-enhancer of activated B cells (NF-κB) and mitogen-activated protein kinase (MAPK) inflammatory pathways, oxidative stress, lipid metabolism, endothelial function, and mitochondrial homeostasis [8].

Estrogens exert cardioprotective effects by increasing nitric oxide (NO) bioavailability, reducing reactive oxygen species (ROS) production, and modulating inflammatory responses, which may partially explain differences in the progression of atherosclerosis and HF between women and men [8].

It has also been demonstrated that the expression of numerous cardiovascular biomarkers and non-coding RNAs (ncRNA) is sex-dependent. MiR-21, associated with fibrosis and cardiac remodeling, exhibits distinct expression profiles in women and men with HF and ischemic heart disease. Similarly, the lncRNA MALAT1, involved in the regulation of angiogenesis, apoptosis, and inflammatory responses, is subject to estrogen-dependent hormonal modulation [9].

Sex-related differences have additionally been observed in inflammatory and metabolic markers such as CRP, troponins, and lipid profiles, which may influence the accuracy of conventional cardiovascular risk stratification models [8].

The importance of sex-related differences becomes particularly relevant in the context of AI and ML models applied in precision medicine. Many AI algorithms are trained on unbalanced clinical datasets in which women remain underrepresented, potentially reducing diagnostic performance in female populations and perpetuating existing clinical inequalities [10].

Studies investigating ML models in acute coronary syndromes demonstrated that the significance of risk predictors and biomarker profiles may differ between sexes, emphasizing the need for explainable and demographically representative AI frameworks [11].

The integration of sex-aware multi-omic profiling may therefore improve prediction accuracy, therapeutic personalization, and preventive strategies in cardiovascular medicine [9].

### 1.5. Economic and Infrastructural Barriers to the Clinical Translation of Multi-Omics and AI Technologies

Despite the growing potential of multi-omics and AI in precision cardiovascular medicine, substantial economic and infrastructural barriers continue to limit their implementation in low- and middle-SDI countries [12].

High-throughput sequencing technologies, advanced proteomic and metabolomic platforms, and AI-driven computational analyses require considerable financial investment, specialized laboratory infrastructure, and access to high-performance computing resources [13].

In many resource-limited settings, restricted access to sequencing facilities, cloud computing technologies, and standardized bioinformatic pipelines significantly hampers the integration of these approaches into routine clinical practice [14].

Additionally, the shortage of trained bioinformaticians and interdisciplinary specialists represents another major obstacle to the effective interpretation and clinical translation of multi-omic data [13].

Importantly, global inequalities in data generation and healthcare infrastructure may also contribute to demographic and geographic biases in AI models. Most large-scale cardiovascular datasets originate from high-income countries, while populations from low-resource regions remain underrepresented [15].

Consequently, AI algorithms trained on such datasets may demonstrate reduced generalizability and lower predictive performance in diverse populations, potentially exacerbating existing disparities in cardiovascular care [15].

Addressing these challenges will require international collaboration, development of cost-effective analytical platforms, open-access bioinformatic resources, and the establishment of globally representative datasets to ensure equitable implementation of precision cardiovascular medicine [15].

## 2. Search Strategy and Selection Criteria

Data for this review were identified through a comprehensive search of electronic databases, including PubMed, Scopus, and Google Scholar, using a combination of keywords such as multi-omics integration, AI, cardiovascular disease, and molecular pathophysiology to capture literature published between 1990 and 2026. While the chronological range encompasses three decades, the selection process was strictly weighted toward the most recent advancements from 2020 to 2026 to ensure the review reflects the current “omics revolution” and the latest state-of-the-art AI-driven diagnostic tools. Inclusion criteria prioritized high-impact global burden studies, recent clinical trials, and pivotal methodological papers describing the shift from simple linear models to sophisticated DL architectures. A limited number of seminal historical publications were retained only where they provide the indispensable foundation for understanding non-coding RNA networks and established biomarkers, which remain essential to current research. Exclusion criteria were rigorously applied to ensure data integrity; specifically, studies were excluded if they featured insufficient detail regarding bioinformatic pipelines, lacked independent cohort validation, or were not published in English. Additionally, reports with excessively small sample sizes that failed to provide statistically robust results, or those focusing on redundant molecular targets without novel mechanistic insights, were omitted. The final evidence base was synthesized into a cutting-edge overview that focuses on the predictive power of emerging technologies and the critical steps required for their successful clinical translation into precision cardiovascular medicine.

## 3. Molecular Mechanisms and Pathways Underlying CVD

### 3.1. Interconnected Molecular Pathways of Inflammation, Oxidative Stress, and Metabolic Dysregulation in CVD

CVDs are characterized by intricate molecular mechanisms that integrate inflammation, oxidative stress, and metabolic dysregulation into a converging pathogenic network. Chronic inflammation is a foundational contributor to atherogenesis and adverse cardiac remodeling, orchestrated through pro-inflammatory signaling pathways and immune cell activation. Inflammatory mediators such as tumor necrosis factor α (TNF-α), interleukin-1β (IL-1β), and other cytokines promote endothelial dysfunction and recruit leukocytes to the vascular intima, while persistent low-grade inflammation amplifies oxidative stress and metabolic imbalance, reinforcing disease progression across multiple cardiovascular phenotypes [16].

Oxidative stress arises when ROS generated from mitochondria, nicotinamide adenine dinucleotide phosphate (NADPH) oxidases (NOX), xanthine oxidase, and uncoupled NO synthase surpass cellular antioxidant defenses, leading to macromolecular damage and activation of maladaptive signaling. Excessive ROS not only directly damage lipids, proteins, and DNA, but also potentiate inflammatory cascades and impair NO bioavailability, exacerbating endothelial dysfunction and contributing to hypertension and atherosclerotic lesion development [17]. Mechanistically, oxidative stress triggers redox-sensitive pathways that intersect with key mediators of inflammation and cell death, including NF-κB and inflammasome activation, thus perpetuating vascular injury and maladaptive remodeling [1].

At the intracellular level, these systemic processes are governed by a complex interplay of signaling kinases and transcriptional regulators. The pro-inflammatory response is primarily driven by the IKKβ (Inhibitor of nuclear factor kappa-B kinase subunit beta) complex, which phosphorylates IκB, allowing NF-κB to translocate to the nucleus and induce cytokine production [18]. Parallelly, stress-activated protein kinases, such as c-Jun N-terminal kinase (JNK) and MAPK, are activated by ROS and TNF-α, contributing to insulin resistance and apoptotic signaling in cardiomyocytes [19]. Conversely, the PI3K/Akt pathway serves as a critical survival signal, though its dysregulation can lead to maladaptive cardiac hypertrophy [20].

On the metabolic front, the AMPK (AMP-activated protein kinase) pathway acts as a central energy sensor; its activation promotes fatty acid oxidation and glucose uptake, offering a protective mechanism against metabolic stress. This is closely linked to the activity of Sirtuin 1 (SIRT1) and Peroxisome proliferator-activated receptor gamma coactivator 1-alpha (PGC-1α), which together regulate mitochondrial biogenesis and antioxidant defenses. The downregulation of the SIRT1/PGC-1α axis is a hallmark of mitochondrial dysfunction in CVDs, linking nutrient sensing to the oxidative damage described above [21].

Complementing these intracellular events, systemic metabolic dysregulation—characterized by insulin resistance, dyslipidemia, and adipose tissue dysfunction—interacts with inflammation and oxidative stress to drive CVD progression. In conditions such as obesity and metabolic syndrome, hypertrophic adipocytes and activated macrophages secrete pro-inflammatory cytokines and free fatty acids, which amplify ROS production and foster a persistent inflammatory milieu [22]. This integrated dysmetabolic state not only impairs systemic glucose and lipid homeostasis but also drives endothelial dysfunction, promotes plaque progression, and predisposes to myocardial dysfunction.

Omics technologies have elucidated mechanistic insights into these interconnected pathways by identifying molecular signatures that reflect the underlying biology of CVD. For example, metabolomic profiles associated with cardiovascular events reveal dysregulated lipid and amino acid metabolism, implicating pathways such as glycerophospholipid metabolism, citrate cycle, and glutathione metabolism in disease progression and severity [23]. Such multi-omic observations support the notion that perturbations across metabolic and redox networks are central to CVD development, and that integrative molecular profiling can capture these perturbations with greater sensitivity and mechanistic relevance than conventional biomarkers.

Collectively, inflammation, oxidative stress, and metabolic dysregulation constitute a tightly interconnected network of pathological processes that underlie the initiation and progression of CVDs. By capturing the molecular complexity of these pathways, omics-derived biomarkers offer a more nuanced framework for risk stratification and precision diagnostics, bridging the translational gap between mechanistic understanding and clinical application.

### 3.2. Modulation of Cardiovascular Pathways by miRNAs, lncRNAs, and Exosomes

NcRNAs, including miRNAs and lncRNAs, have emerged as critical regulators of cardiovascular molecular pathways. These molecules operate within a multilayered regulatory network that modulates gene expression and cell signaling. This system is further integrated with exosome-mediated RNA transfer, which facilitates intercellular communication and provides a stable source of mechanistically relevant biomarkers for CVD [24].

### 3.3. LncRNAs as Epigenetic Regulators

LncRNAs, defined as transcripts longer than 200 nucleotides with limited protein-coding capacity, represent another major class of regulatory RNAs that influence CVD mechanisms. LncRNAs modulate gene expression through diverse mechanisms, including chromatin remodeling, transcriptional control, and acting as competing endogenous RNAs that sequester miRNAs. Recent comprehensive reviews indicate that lncRNAs such as LIPCAR, MALAT1, and others are implicated in processes including cardiac hypertrophy, fibrosis, and HF, often by interacting with molecular pathways governing inflammation and stress responses [25,26]. Furthermore, functional analyses have revealed that lncRNAs participate in the regulation of oxidative stress pathways, suggesting their involvement in redox homeostasis and antioxidant responses, which are central to CVD pathophysiology [27]. As stable regulatory molecules, lncRNAs constitute a critical layer of the cardiovascular epigenome, serving as potential targets for therapeutic intervention and precision diagnostics.

### 3.4. Exosomal Signal Trafficking and Intercellular Communication

Exosomes are nanoscale, membrane-bound extracellular vesicles formed within endosomal multivesicular bodies and released into the extracellular space, enabling the transfer of biomolecules such as proteins, lipids, and nucleic acids, including miRNAs and lncRNAs, between cells. These vesicles are secreted by virtually all cell types relevant to the cardiovascular system—including endothelial cells, cardiomyocytes, smooth muscle cells, fibroblasts, immune cells, and stem cells—and constitute a fundamental mechanism of intercellular communication that influences cardiovascular homeostasis and disease progression [28].

The specificity and diversity of exosomal cargo reflect the physiological or pathological state of the donor cells, enabling precise signaling to recipient cells. Exosomes can be internalized by target cells via endocytosis, direct fusion with the plasma membrane, or receptor-mediated interactions, thereby delivering their cargo into the cytosol and modulating gene expression and cellular function [29]. Such cargoes include regulatory RNAs (miRNAs, lncRNAs, circular RNAs (circRNAs)), signaling proteins, and other effectors that can trigger or inhibit pathways linked to inflammation, oxidative stress, fibrosis, angiogenesis, and metabolic regulation, positioning exosomal signaling as a key mediator of cardiovascular pathophysiology [28].

Exosomal communication operates through paracrine, autocrine, and endocrine routes, enabling both local and systemic effects. Paracrine exosome signaling contributes to cellular crosstalk within the cardiac microenvironment, such as between endothelial cells and vascular smooth muscle cells (VSMCs) or between cardiomyocytes and fibroblasts, modifying processes like endothelial function, vascular remodeling, and cardiac hypertrophy [30]. Endocrine exosomal signaling also facilitates communication between distant tissues and the heart, exemplifying how systemic stressors or metabolic conditions can influence cardiovascular outcomes via circulating exosomes [31].

In pathophysiological contexts, exosomal RNAs have been shown to both propagate and mitigate disease processes. For instance, exosome cargoes carrying pro-inflammatory miRNAs can exacerbate endothelial dysfunction and atherosclerotic progression, while exosomes released from stem cells or progenitor cells enriched with cardioprotective miRNAs can promote angiogenesis and attenuate ischemic injury [32]. The dual nature of exosomal signaling—capable of both aggravating and ameliorating pathology depending on cargo composition and cellular origin—underscores the complexity of intercellular communication in CVD and provides a mechanistic rationale for the development of exosome-based diagnostics and therapeutics.

Given their stability in circulation and ability to reflect cell-specific molecular changes, exosomes and their molecular cargo are increasingly recognized not only as mediators of intercellular communication but also as promising biomarkers for disease diagnosis and progression. Moreover, engineered exosomes are under investigation for targeted delivery of therapeutic nucleic acids and small molecules, highlighting their potential as a translational platform in precision cardiovascular medicine [33].

### 3.5. MiRNA Biogenesis and Gene Regulation Networks

MiRNAs are small, but still significantly vital non-coding RNAs that influence gene expression by repressing or degrading mRNA translation into proteins [34]. These small molecules are initially transcribed from DNA to a primary miRNA (pri-miRNA), cleaved into double-stranded RNA containing from 18 to 23 nucleotides (precursor miRNA, pre-miRNA), and then processed into a single-stranded complex (miRNA plus protein) that binds to mRNA [35]. MiRNAs can be functionally categorized into two primary clusters based on their regulatory roles. The first group, often referred to as oncomiRs in the context of disease, negatively regulates gene expression by binding to the 3′UTRs of target mRNAs, leading to their degradation or translational inhibition. This process controls critical biological functions, including cell proliferation, growth, and migration, while the second group acts as tumor suppressors by maintaining genomic stability and regulating DNA repair and apoptosis. MiRNAs have been shown to affect mRNA stability by halting translation or by causing its degradation through base pairing with mRNAs, acting as negative regulators of protein translation and, therefore, playing key roles in controlling biological cell function and communication [36]. This regulatory capacity allows miRNAs to influence a broad array of biological processes relevant to CVD, such as endothelial function, cardiomyocyte survival, fibrosis, inflammation, and oxidative stress response. MiRNAs, produced by all cell types, and the same miRNA may be derived from various cells, such as endothelial cells, macrophages, VSMC, thrombocytes, and eventually are secreted in blood plasma [37]. Thus, miRNAs have been at the center of scientific attention as a potential target of treatment in inflammatory diseases, such as cardiovascular diseases or neoplasms. Some researchers believe that miRNAs could be used as highly sensitive early diagnostic biomarkers and therapeutic targets for many CVDs such as atherosclerosis, acute myocardial infarction (AMI), HF, hypertension, and stroke impaired miRNAs expression has been widely described in condition of cardiomyopathy, atrial fibrillation, atrial hypertension, metabolic syndrome, stroke [38], and processes as angiogenesis, inflammation and cardiac remodeling, which are central to the development and progression of CVDs [39]. Studies have demonstrated that the expression of numerous miRNAs is altered in cardiovascular conditions, and that these miRNAs also exert regulatory control over oxidative stress and inflammation.

The most abundant miRNAs expressed in myocardial tissue and involved in cardiomyocyte differentiation in the early stages of heart development are *miR-1*, *miR-133a*, *miR-208*, and *miR-499* [40]. Specific cardiac-enriched miRNAs, such as miR-1 and miR-133, are associated with muscle differentiation and remodeling, while others, such as miR-208, regulate cardiac contractile protein expression and hypertrophic responses [24]. Several miRNAs have been shown to modulate ROS-related pathways and antioxidant defenses, thereby influencing redox balance in cardiovascular tissues. Atherosclerosis advancement is related to several miRNAs, including *miR-15a-5p*, *miR-199a-3p*, *miR-34a*, *miR-146a*, and *miR-217* [41], contributing to endothelial dysfunction and inflammation. Experimental evidence indicates that dysregulated miRNA expression can exacerbate oxidative damage and the production of inflammatory mediators, underscoring their mechanistic relevance in CVD progression [42]. Moreover, integrated reviews have highlighted the involvement of miRNAs in atherosclerosis and related metabolic conditions, suggesting that these small non-coding RNAs serve as functional links between metabolic stress, chronic inflammation, and vascular pathology [43].

*miR-21* levels were significantly elevated in patients with AMI compared to those with angina pectoris or healthy people, indicating that plasma *miR-21* molecule may be a novel biomarker for the diagnosis of this condition [44]. MiRNAs have been extensively studied in a number of different human cancers, acting both as oncogenes and tumor suppressors. For instance, *miR-21* can promote the progression of lung cancer through multiple signaling pathways, with a focus on PI3K/AKT, MEK/ERK, TGF-β/*SMAD*, Hippo, NF-κB, and STAT3 signaling pathways. Most studies have shown that *miR-21* acts as an oncogene in lung cancer and that high expression of *miR-21* is associated with poor prognosis for patients with lung cancer, due to increased drug resistance [45].

While these non-coding RNA mechanisms offer a window into the dynamic and real-time regulation of cardiovascular health, the fundamental blueprint of individual susceptibility to CVD remains encoded in the genome, a domain explored in the following section.

## 4. Emerging Biomarkers in Circulation

### 4.1. Genomics

Genetics contributes to the risk of most CVDs. Although much remains to be learned, common CVDs are generally considered multifactorial, involving many genes and environmental influences. In contrast, genetic testing is clinically recommended for certain single-gene cardiovascular disorders. About 15% of MIs occur due to familial factors independent of traditional risks. Since the 1990s, research has expanded rapidly through family linkage studies, candidate gene analyses, and genome-wide association studies (GWAS). One major finding identified a region at 13q12–13 associated with myocardial infarction (MI) and later linked it to variants in the arachidonate 5-lipoxygenase-activating protein (ALOX5AP) gene, which is associated with increased inflammation. This association has been observed in several populations, including British, Icelandic, and Scottish cohorts, as well as a small U.S. group with acute coronary syndrome. GWAS identified the 9p21.3 locus as a significant MI/coronary artery disease (CAD) marker, a result repeatedly confirmed since. This region contains the noncoding RNA ANRIL—Antisense Non-coding RNA in the INK4 Locus, which is being studied for its role in atherosclerosis. More recent large-scale GWAS from the CARDIoGRAM—Coronary Artery Disease Genome-wide Replication and Meta-analysis consortium identified additional loci, many of which are not connected to traditional CAD risk factors, suggesting involvement of previously unknown biological pathways. However, these known variants explain only a small part of CAD heritability, indicating many remain undiscovered. Only a few genes affecting LDL-C, such as proprotein convertase subtilisin/kexin type 9 (PCSK9) and apolipoprotein E (APOE) gene, have shown strong links to MI/CAD [46].

PCSK9 was identified in the early 2000s as the third locus associated with familial hypercholesterolemia. Gain-of-function mutations in PCSK9 cause autosomal dominant hypercholesterolemia, whereas loss-of-function variants are associated with markedly reduced LDL-C levels and lower coronary artery disease risk. Experimental studies demonstrated that PCSK9 regulates hepatic LDL receptor degradation, thereby influencing plasma cholesterol levels. Structurally, PCSK9 consists of a pro-domain, catalytic domain, and C-terminal cysteine-rich domain, with mutations in these regions affecting LDL receptor interaction and lipid metabolism. Hepatic PCSK9 expression is regulated by sterol-responsive transcription factors, including SREBP and HNF1. The discovery of PCSK9 enabled the development of targeted therapies, including monoclonal antibodies and siRNA-based agents, which effectively lower LDL-C and reduce ASCVD risk [47].

Lipoprotein(a) (Lp(a)) is a genetically determined cardiovascular risk factor primarily regulated by variants in the LPA gene, which encodes apolipoprotein(a). Polymorphisms within the LPA locus influence apo(a) structure and expression, including the number of kringle IV repeats, leading to marked interindividual variability in plasma Lp(a) concentrations. Genome-wide studies have identified numerous LPA variants strongly associated with elevated Lp(a) levels and increased risk of coronary heart disease, myocardial infarction, stroke, and aortic valve stenosis. Genetic evidence indicates that these variants increase cardiovascular risk mainly through higher circulating Lp(a) particle concentrations. Because Lp(a) levels are almost entirely genetically regulated, conventional lipid-lowering therapies, including statins, have limited effects, while PCSK9 inhibitors provide only modest reductions. In contrast, emerging RNA-based therapies targeting hepatic apo(a) synthesis have shown substantial Lp(a)-lowering efficacy, although their long-term clinical benefits remain under investigation [48].

The titin gene (TTN), encoding titin—the largest human protein and a key structural component of striated muscle—is a major genetic determinant of cardiomyopathies. Advances in next-generation sequencing enabled comprehensive analysis of this large and complex gene, revealing numerous pathogenic variants, predominantly located within exonic regions. Truncating TTN variants, particularly those affecting the A-band region, are strongly associated with DCM and represent one of its most common genetic causes. These variants disrupt normal titin protein production and function, contributing to myocardial remodeling and dysfunction. In contrast, TTN variants linked to hypertrophic, restrictive, or arrhythmogenic cardiomyopathies are less frequent, and their pathogenic significance remains less clearly established. The clustering of pathogenic variants within highly conserved TTN exons further supports their central role in cardiomyopathy development [49].

A large next-generation sequencing study in patients with DCM demonstrated that rare pathogenic variants are present in approximately one-third of cases, with TTN variants being the most frequent. The clinical significance varied according to the affected gene. Variants in the lamin A/C gene (LMNA) and desmosomal genes were associated with a substantially increased risk of ventricular arrhythmias and sudden cardiac death, even in the absence of severe left ventricular dysfunction, highlighting the importance of genotype-specific risk stratification. Although overall survival did not significantly differ between carriers and non-carriers, pathogenic variants were generally associated with less favorable clinical outcomes. These findings support the growing role of genetic testing in prognostic assessment and personalized management of DCM [50].

A 2020 study investigating vascular smooth muscle cell (VSMC) phenotypes identified several genetic loci associated with processes involved in atherosclerosis, including calcification, proliferation, and migration [6]. Among these, the 1q41 locus containing the MIA3 gene was linked to reduced VSMC proliferation and lower MIA3 expression, suggesting a potential role in plaque vulnerability and fibrous cap stability. Additional CAD-associated loci, including 9p21.3, ADAMTS7, TCF21, PHACTR1, GUCY1A3, and PLPP3, were connected to pathways regulating VSMC differentiation, migration, NO signaling, and mechano-transduction [51].

Genomic studies in cardiovascular disease have demonstrated that pathogenic variants can be identified long before clinical manifestation, enabling pre-symptomatic risk prediction. Variants in genes such as PCSK9, LPA, TTN, LMNA, and desmosomal genes are associated with increased susceptibility to coronary artery disease, dilated cardiomyopathy, and arrhythmic complications. However, although genetic markers are valuable for assessing disease susceptibility, their ability to predict disease severity or therapeutic response remains limited because the genome itself is static and does not reflect dynamic disease progression.

### 4.2. Transcriptomics

Transcriptomics focuses on analyzing all RNA molecules present in cells or biological fluids, offering valuable insight into mechanisms driving CVD. Although most human genes are transcribed into RNA, only a small fraction encodes proteins, highlighting the widespread regulatory role of non-coding RNAs such as miRNAs and lncRNAs. These molecules are key regulators of cardiovascular development, aging, and disease processes. Because RNA networks involve complex interactions among coding transcripts, regulatory RNAs, proteins, and post-transcriptional modifications, transcriptomic approaches provide a systems-level view that can support personalized and even sex-specific diagnosis, prognosis, and therapy in cardiovascular medicine [52].

Advances in single-cell transcriptomics have transformed the understanding of cardiovascular development and disease by enabling transcriptomic profiling at the individual cell level. Unlike bulk RNA sequencing, scRNA-seq reveals cellular heterogeneity, lineage transitions, and disease-associated molecular states within cardiac tissues. More recent spatial transcriptomics approaches integrate gene expression with positional tissue context, overcoming the loss of spatial information caused by cell dissociation. ScRNA-seq has been used to map developmental trajectories of cardiac cell populations, identify state-specific gene expression changes, and uncover previously unrecognized subpopulations involved in processes such as valve formation, endothelial maturation, fibrosis, and cardiomyocyte remodeling under stress. Studies applying scRNA-seq in congenital heart disease have demonstrated how altered transcriptional signaling in specific lineages contributes to abnormal heart formation. Technical challenges remain, including incomplete transcript capture, loss of spatial information, and varying throughput requirements, but ongoing improvements in sequencing chemistry and computational analysis continue to expand the power of transcriptomic profiling in cardiovascular research [53].

MiRNAs constitute an important component of cardiovascular transcriptomic profiling and are increasingly investigated as indicators of disease-specific molecular signatures. More detailed aspects of miRNA biogenesis, regulatory mechanisms, and their role in cardiovascular pathology have been discussed in the Section 3. Within transcriptomic studies, miR-21 remains one of the most frequently analyzed miRNAs because its altered expression has been consistently observed across multiple cardiovascular conditions, including myocardial infarction, heart failure, hypertension, and atherosclerosis. Owing to its reproducible detection in circulating samples, miR-21 has attracted considerable interest as a potential biomarker and therapeutic target, although its clinical applicability is still limited by tissue-specific and context-dependent expression patterns [54].

Other cardiac-associated miRNAs, particularly miR-1, miR-133a, miR-208, and miR-499, are also widely studied in cardiovascular transcriptomics due to their strong association with myocardial injury and cardiac tissue remodeling. Their expression profiles have been investigated in acute MI and sudden cardiac death (SCD), where distinct patterns may reflect underlying molecular and electrophysiological alterations. In particular, miR-1 and miR-499 demonstrated high sensitivity in distinguishing SCD from AMI, whereas miR-208 showed high specificity for AMI. These observations support the growing value of circulating miRNA signatures in transcriptomic-based cardiovascular diagnostics and risk stratification, although additional validation studies are still necessary before routine clinical implementation [55].

In cardiovascular transcriptomic studies, lncRNAs such as MALAT1 have gained considerable attention, while their broader regulatory mechanisms and roles in cardiovascular pathology were discussed in the Section 3. MALAT1 expression is increased in response to cardiovascular stressors such as hypoxia, oxidative stress, hyperglycemia, and inflammation, particularly in endothelial cells and cardiomyocytes. It regulates processes including apoptosis, angiogenesis, inflammation, fibrosis, lipid accumulation, and cardiac remodeling through interactions with miRNAs and downstream signaling pathways. Dysregulated MALAT1 expression has been associated with atherosclerosis, myocardial infarction, diabetic cardiomyopathy, pulmonary hypertension, and cardiac fibrosis. In addition to its mechanistic role in cardiovascular pathophysiology, circulating MALAT1 has been proposed as a potential biomarker, particularly in acute MI [56].

H19 is a highly conserved lncRNA enriched in cardiac and vascular tissues, with low levels in adulthood but retained activity in the heart. It is involved in cardiovascular pathophysiology, with altered expression reported in conditions such as atherosclerosis, CAD, MI, ischemia/reperfusion injury, and HF. In MI, H19 expression increases shortly after injury and may act as a compensatory mechanism, but its effects depend on timing and cell type. Early overexpression in cardiac fibroblasts may worsen fibrosis, while later restoration of H19 can improve cardiac function by reducing cell death and supporting autophagy. Similar time-dependent expression patterns are observed in pressure-overload and HF models, where loss of H19 in later disease stages correlates with worsening cardiac function. H19 also contributes to cardiac remodeling, with some studies showing protective effects against hypertrophy and others linking H19 overexpression to cardiomyocyte apoptosis, depending on disease model and regulatory pathways engaged. Overall, evidence suggests that H19 may contribute to cardiovascular disease progression and has potential as both a biomarker and therapeutic target, although its precise mechanisms remain incompletely defined and require further investigation [57].

CircRNAs are a stable class of non-coding RNAs formed through covalent joining of their 3′ and 5′ ends. In the cardiovascular system, they regulate cardiomyocyte proliferation, survival, and responses to oxidative stress, particularly after MI. Some circRNAs localize to mitochondria, where they control mitochondrial function, membrane potential, and production of ROS, making them promising therapeutic targets. One example is circSamd4, a mitochondria-localized circRNA highly expressed in neonatal cardiomyocytes. Its overexpression reduces mitochondrial ROS, prevents mitochondrial permeability transition pore opening, and limits oxidative DNA damage. As a result, circSamd4 promotes cardiomyocyte cell-cycle re-entry, decreases apoptosis, and enhances heart regeneration after MI. Examinations suggest that circSamd4 is a potential therapeutic target for post-MI HF [58].

Transcriptomics examines all RNA molecules in cardiovascular tissues and fluids, providing insight into disease mechanisms and enabling dynamic biomarkers that change with disease progression and treatment response. Non-coding RNAs such as miRNAs, lncRNAs, and circRNAs show disease-specific expression patterns and regulate key processes like fibrosis, oxidative stress, and cardiomyocyte survival, making them promising diagnostic and prognostic markers. However, clinical use is still limited by variability in sample handling and the lack of standardized measurement methods.

### 4.3. Proteomics

Advances in high-throughput proteomic technologies now allow simultaneous measurement of hundreds of plasma proteins, enabling a deeper understanding of mechanisms driving cardiovascular mortality in chronic CHD. Established biomarkers such as NT-proBNP, high-sensitivity troponin T, CRP, and cystatin-C remain strong predictors of cardiovascular death. Large studies using multiplex assays, including proximity extension analysis, have identified additional proteins linked to key pathological processes such as myocardial stress and fibrosis (e.g., BNP, sST2, SPON1), cell death and apoptosis (e.g., TNF-R1, TRAIL-R2), renal dysfunction (e.g., FGF-23, TIM-1), inflammation and oxidative stress (e.g., IL-6, GDF-15, OPG), and vascular remodeling (e.g., HGF). These findings highlight that proteomic profiling can uncover multiple independent pathways contributing to risk, support the discovery of new therapeutic targets, and improve individualized treatment strategies in patients with chronic CHD [59].

Cardiac troponin is the gold standard for detecting AMI, especially by identifying a rise or fall in its levels. High-sensitivity assays can detect cTn even in healthy individuals, and elevated cTn is also observed in chronic cardiovascular and systemic conditions, including HF, CAD, diabetes, and chronic kidney disease. In these settings, troponin release may reflect mechanisms beyond cell necrosis, including apoptosis, increased membrane permeability, or normal cell turnover. As detection methods advance, cTn may support more personalized risk assessment and provide deeper diagnostic information [60]. The discovery of ANP and later BNP revealed that the heart also functions as an endocrine organ. BNP and NT-proBNP are produced from the same precursor encoded by the NPPB gene and released mainly by ventricular cardiomyocytes in response to pressure or volume overload. BNP is biologically active and promotes vasodilation, diuresis, and natriuresis, while NT-proBNP is inactive and cleared mainly through the kidneys. Their blood levels are low in healthy individuals but rise significantly in HF, making them core biomarkers for diagnosis and risk assessment recommended by major clinical guidelines. NT-proBNP has a longer half-life and higher circulating concentrations than BNP due to slower clearance. Although helpful for detecting early cardiac dysfunction, their interpretation must take into account factors such as age, renal function, arrhythmias, and obesity. Additional biomarkers, including high-sensitivity troponins and sST2, may complement BNP and NT-proBNP for improved prognostic assessment, and multi-marker approaches are a future direction of research [61].

Galectin-3 is a glycan-binding protein that plays a key role in inflammation, fibrosis, and tissue remodeling and is increasingly recognized as a biomarker and potential therapeutic target in many CVDs. Its expression rises in acute and chronic heart conditions such as atherosclerosis, acute coronary syndromes, MI, HF, hypertrophic cardiomyopathy, atrial fibrillation, and hypertension. Galectin-3 promotes macrophage activation, foam cell formation, vascular smooth muscle proliferation, and collagen deposition, driving adverse structural changes in the heart and vessels. Elevated circulating levels are associated with higher disease severity, worse prognosis, and increased mortality. While it initially may have protective effects, prolonged elevation contributes to fibrosis and harmful remodeling. It is also being explored as a therapeutic target, with experimental studies suggesting that its inhibition may reduce fibrosis and improve cardiac function. In addition, its integration with established biomarkers such as NT-proBNP and cardiac troponins may improve cardiovascular risk stratification [62].

Growth differentiation factor-15 (GDF-15) is a stress-responsive cytokine from the TGF-β family, normally present at low levels but strongly upregulated by tissue injury, inflammation, and cardiac stress. Experimental studies suggest it has cardioprotective effects, limiting cell death and adverse remodeling after ischemia. Clinically, elevated circulating GDF-15 is consistently associated with increased risk of cardiovascular events and mortality across a range of conditions, including acute coronary syndromes, stable CAD, and HF. In heart failure, GDF-15 reflects disease severity and provides prognostic information complementary to natriuretic peptides, while in other settings, such as atrial fibrillation, it predicts mortality and bleeding risk, and they are associated with worse outcomes and higher HF risk in chronic kidney disease. Overall, GDF-15 is a promising biomarker reflecting inflammation and tissue injury, but its routine clinical use still requires further validation [63].

Proteomic research enables simultaneous assessment of many circulating proteins, offering insight into multiple biological pathways that contribute to cardiovascular risk in chronic coronary disease. Established markers such as NT-proBNP and hs-troponin remain strong predictors of mortality, while high-throughput assays have identified additional proteins linked to myocardial stress, fibrosis, inflammation, apoptosis, renal dysfunction, and vascular remodeling. Biomarkers like BNP/NT-proBNP, troponins, galectin-3, and GDF-15 provide information closely aligned with the disease phenotype, improving risk stratification and supporting individualized treatment. However, a key limitation of proteomic biomarkers is their low specificity—many of these proteins also change in non-cardiac or systemic diseases, which complicates interpretation.

### 4.4. Metabolomics

Metabolomics offers a powerful way to study the metabolic disturbances underlying CVD, including HF. Because HF is a complex, multifactorial condition—often occurring in older patients with multiple comorbidities—metabolic profiling can help clarify disease mechanisms and identify biomarkers linked to prognosis. Changes in circulating ceramides, amino acids, acylcarnitines, and organic acids reflect shifts in energy and amino acid metabolism and have been associated with HF severity and outcomes. Although metabolite-based risk scores such as the Prognostic Metabolic Profile and the Cardiac Lipid Panel show promise, none have yet entered clinical practice due to limited standardization and a lack of comparison with established clinical tools. A major barrier to translation is the absence of consistent protocols for sample handling, analysis, data processing, and reporting. Despite these challenges, recent meta-analyses have identified several metabolites—some protective, others linked to worse prognosis—highlighting metabolomics as a promising, though still emerging, field for improving understanding and risk assessment in CVD [64].

Metabolomic studies have identified medium- and long-chain acylcarnitines as consistently dysregulated in patients with cardiovascular disease and cardiovascular events. These alterations reflect disturbances in carnitine-dependent fatty acid transport and mitochondrial β-oxidation, suggesting underlying metabolic and energetic dysfunction. Elevated circulating levels likely reflect impaired fatty acid oxidation and mitochondrial dysfunction. Importantly, excessive accumulation of long-chain acylcarnitines may also directly affect cardiac performance by disrupting ion balance, destabilizing the action potential, and impairing mechanical and electrical activity. Because the heart is a major source of circulating long-chain acylcarnitines—even in the absence of overt myocardial injury—these metabolites may indicate early metabolic stress before conventional cardiac biomarkers such as hs-cTnT or NT-proBNP become detectable. This makes them promising candidates for early risk stratification in CVD. However, their clinical applicability remains limited by the need for further mechanistic studies and validation in large, diverse populations [23].

The gut microbiome has emerged as an important contributor to cardiovascular disease, with trimethylamine N-oxide (TMAO) being one of the most studied metabolites. TMAO is generated through gut microbial metabolism of dietary choline, betaine, and L-carnitine, followed by hepatic oxidation. Elevated circulating TMAO levels have been associated with gut dysbiosis and increased cardiovascular risk, including atherosclerosis and vascular inflammation. Mechanistically, TMAO impairs endothelial NO signaling, activates PKC and NF-κB pathways, and stimulates the release of inflammatory cytokines, promoting vascular inflammation. It also contributes to atherosclerosis by inhibiting reverse cholesterol transport, increasing *PCSK9* levels, enhancing macrophage scavenger receptor expression, and raising platelet reactivity. These actions collectively favor plaque progression and thrombosis. Despite strong experimental support, human data are still insufficient to confirm a direct causal relationship between TMAO and CVD. Therapeutic approaches aimed at reducing TMAO production are under investigation, but further clinical studies are required to determine whether TMAO can serve as a robust biomarker of gut dysbiosis and cardiovascular risk [65].

Branched-chain amino acids (BCAAs)—leucine, isoleucine, and valine—are essential metabolites derived from diet and gut microbial activity. Beyond their role in protein synthesis, altered BCAA metabolism has been associated with metabolic disorders (obesity, insulin resistance, and type 2 diabetes) and cardiovascular diseases, including HF, CAD, hypertension, and arrhythmias. Impaired BCAA catabolism can lead to their accumulation in circulation, reflecting systemic metabolic dysfunction. Elevated circulating BCAA levels have been linked in several studies to adverse cardiovascular outcomes, although associations may vary depending on disease stage and metabolic context. Therapeutically, targeting BCAA metabolism—through dietary restriction or drugs that enhance BCAA oxidation—offers potential for future CVD treatment, but clinical validation is still needed [66].

Ketone bodies, mainly acetoacetate and β-hydroxybutyrate, are energy substrates produced by the liver during metabolic stress such as fasting, exercise, or altered nutrient availability. They serve as an alternative fuel for the heart, which is metabolically flexible and can oxidize ketones when glucose or fatty acid utilization is impaired. In HF, this flexibility becomes critical. As fatty acid and glucose oxidation decline in hypertrophied and failing hearts, ketone utilization increases, supported by higher circulating ketone levels and upregulation of ketolytic enzymes. Evidence from human and animal studies shows that the failing heart consumes more ketones and that genetic loss of ketone-oxidation enzymes worsens cardiac dysfunction. Conversely, ketone supplementation or ketone-enhancing diets can reduce adverse remodeling and improve systolic function in several preclinical HF models. Beyond cardiomyocytes, ketone bodies also modulate immune, inflammatory, and endothelial pathways, suggesting broader cardioprotective effects. Early clinical findings indicate that higher β-hydroxybutyrate levels may reflect HF severity, though its value as a biomarker requires further study. Overall, increased ketone metabolism appears to be an adaptive response in HF, and enhancing ketone availability is emerging as a promising therapeutic strategy deserving further investigation [67].

Metabolomics provides valuable insights into the metabolic disturbances underlying CVD by identifying changes in key pathways involving lipids, amino acids, and energy metabolism. Medium- and long-chain acylcarnitines, TMAO, branched-chain amino acids, and ketone bodies all reflect distinct metabolic alterations linked to cardiovascular risk, disease progression, or HF severity. Acylcarnitines and TMAO may signal early metabolic stress and gut microbial imbalance, while elevated BCAAs and increased ketone utilization highlight broader systemic and cardiac metabolic shifts. Although these metabolites show promise as biomarkers and potential therapeutic targets, their clinical application remains limited and requires further mechanistic and large-scale validation studies.

### 4.5. Epigenomics

Epigenetic changes can modify gene activity without altering the DNA sequence, mainly through DNA methylation, histone modifications, and RNA-based mechanisms. Because these changes are reversible, they present attractive targets for future therapies. Early studies link these epigenetic processes to CVD, such as atherosclerosis, HF, MI, and cardiac hypertrophy. Although various epigenetic agents are under investigation, none have yet entered clinical trials or received FDA approval for CVD treatment. Lifestyle factors—especially diet and exercise—remain key for CVD prevention. Certain foods, including polyphenols, cocoa, and folic acid, can influence epigenetic pathways, though excessive folic acid intake may increase cancer risk. Some existing treatments, like statins, may already affect epigenetic regulation. NO has long been recognized as important for cardiovascular function, but developing therapies that safely enhance or deliver NO remains challenging. Similarly, non-coding RNAs, including miRNAs and lncRNAs, show promise as biomarkers and potential therapeutic targets, though current findings come mainly from animal studies and technical barriers remain [68].

Aging increases the risk of many diseases, but people age biologically at different rates. DNA methylation is influenced by genetics and environment and has become a key marker of biological aging. Epigenetic clocks—built from DNAm patterns—can estimate biological age and predict health outcomes. Second-generation clocks, such as PhenoAge and GrimAge, incorporate disease-related and mortality-associated features, improving prognostic value beyond earlier models. Large-scale genomic studies have identified multiple genetic loci associated with DNAm-based aging traits, many of which are involved in immune and metabolic pathways and overlap with known determinants of aging-related phenotypes. Shared genetic links were found between epigenetic aging, smoking, obesity, education, and parental lifespan. GrimAge showed especially strong genetic connections with longevity and lung cancer, likely reflecting its incorporation of smoking-related methylation markers. Mendelian randomization suggested causal effects of body fat and smoking behavior on GrimAge acceleration, while higher education appeared protective. Although these biomarkers correlate with many health outcomes, the study found limited evidence that DNAm measures themselves directly cause disease. It also provided the first polygenic risk scores for six epigenetic aging traits. Overall, the findings show that epigenetic clocks reflect a combination of genetic factors, lifestyle, and aging biology, making them valuable indicators of biological aging [69].

CpG sites, particularly within CpG islands, are key regions of DNA methylation that regulate gene expression and have been increasingly associated with cardiovascular disease. Epigenome-wide studies have linked differential CpG methylation patterns to CVD-related processes, including atherosclerosis and coagulation, although findings are not fully consistent across studies due to differences in populations, tissues, and analytical methods. Some CpG sites overlap with those used in epigenetic clocks, supporting a relationship between DNA methylation–based aging and cardiovascular risk. Overall, CpG methylation patterns represent potential biomarkers linking environmental and lifestyle factors to cardiovascular disease, but their clinical application requires further standardization and validation [70]. Although these approaches offer valuable insights, especially into how behavior and environment shape cardiovascular health, epigenetics still has far fewer clinical studies compared with proteomics and transcriptomics, and no epigenetic therapies for CVD have yet reached clinical trials. The summary of biomarkers in circulation is presented in the Table 1.

## 5. Overview of ML Techniques

AI is wrongly associated in the public mind with ChatGPT, image generators, and other Internet sources, which negatively prejudices its use in health care. It is important to remember that there is a variety of branches of AI that are possibly applicable in medical fields. One of which is ML. ML is a subset of AI which aims to enable computer systems to create a model that is capable of making predictions without being programmed by immense sets of data. The algorithm is trained on smaller sets of data that enable it to make predictions outside of the training set. ML exploits statistical theorems in mathematical models because its main task is to detect similarities, patterns, and draw conclusions [71].

ML approaches have already demonstrated substantial diagnostic and predictive utility across multiple medical specialties beyond cardiovascular medicine. In endocrinology, explainable ML models such as the ExtraTreeClassifier have shown high effectiveness in the early detection of type 2 diabetes by integrating demographic, biochemical, and clinical parameters, emphasizing the growing importance of interpretable AI systems in personalized medicine [72].

Similarly, in oncology and medical imaging, self-organizing map (SOM) neural networks (NNs) combined with block-processing techniques have been successfully applied to cancer-zone detection in noisy MRI images, improving diagnostic precision and robustness in complex imaging datasets. These findings highlight the broad translational potential of ML methods across diverse healthcare domains and support their increasing integration into precision diagnostics and predictive medicine [73].

ML algorithms can be divided into three main categories—supervised learning, unsupervised learning, and DL—from which each can serve for different purposes in biomarker discovery and in various medical fields [74]. An overview of these techniques, including their specific algorithms and clinical applications in cardiovascular medicine, is presented in Table 2.

### 5.1. Use of Supervised Learning for Classification

Supervised learning requires a labeled datasets that contain exemplary inputs paired with their correct answers. As these inputs are passed through a machine-learning algorithm, the model continually updates its parameters until it fits the data [75]. This labeled training information serves as the “ground truth,” directly guiding the model to learn how features correspond to their associated labels, enabling algorithms to classify and predict outcomes. The technique is commonly used in observation and classification, applying algorithms such as linear classifiers, support vector machines (SVMs), decision trees, k-nearest neighbors (KNNs), logistic regression, and random forests (RFs) [76].

SVMs are considered to be an effective ML technique for classification. The algorithm exploits kernel solution functions enabling distinguishing between several categories of data by identifying the hyperplane which most effectively establishes the margin of the closest data points of opposite classes (support vectors) [77]. It has to be taken into consideration that multiple hyperplanes can be determined to differentiate categories, enabling SVM to locate the most optimal boundary between datasets. SVM is potent in high-dimensional or non-linear datasets such as microbiome and omics data [78] or cardiac physiology (nonlinear ECG features). However, its limitations include tardy training when using extensive sample sizes or difficulty in scaling comprehensive datasets—proper parameters must be employed for the algorithm to function and determine correct data. If the classifier becomes overly fit, an SVM memorizes the training data instead of learning to generalize [79]. Another limitation is choosing an appropriate kernel function, which implies that the real structure of data must be applied, while other algorithms, such as NNs or RFs, are able to identify the structure automatically. Moreover, SVM may create impediments in data interpretation and understanding due to the weights of the variables [80].

**Figure 1:**
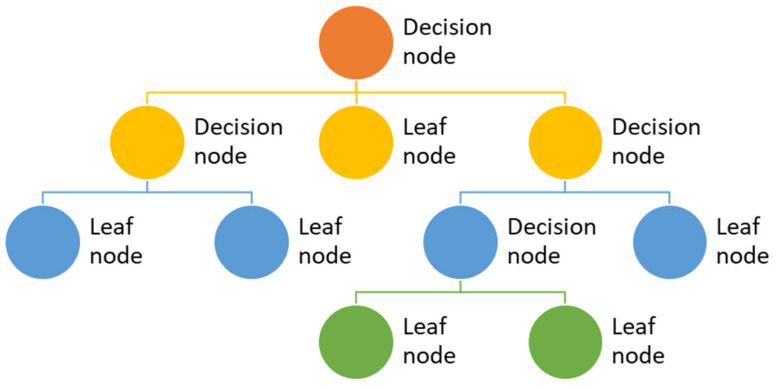
Illustration of how a decision tree works.

RF is another ML model used for classification, which is considered to be a variation of decision trees. In order to gain an understanding of how RF works, one has to understand the algorithm behind decision trees. They are for determining the final classification and are based on the concept that each branch, known as the decision node, represents consecutive steps leading to a leaf node. When splitting a node, the cluttering (impurity) of the node’s data content is expressed as a numerical value called entropy. The node is split to organize its content; that is, the ratio or difference (information gain) of the entropy of the nodes before and after is maximized [81], which is illustrated in Figure 1. With the trees’ gradual growth, the data can be subdivided into separate characteristics. It has to be kept in mind that the more complex the data is, the more interference is incorporated into the characteristics, which may lead to overfitting of the data.

**Figure 2:**
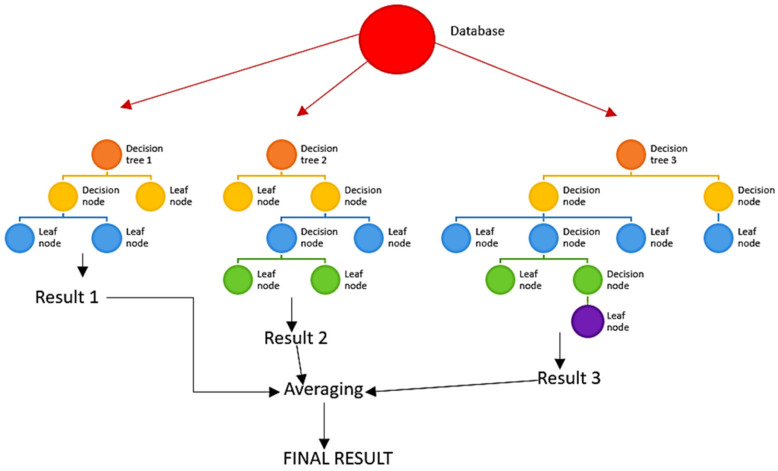
Illustration of how the RF algorithm works.

In order to dispose of the overfitting, RF is applied. Multiple decision trees are created in parallel, incorporating data chosen arbitrarily. However, it does not analyze all possible feature splits, but it only chooses a subset—each tree operates on a different set of data. Moreover, it does not solely rely on bagging, but on both bagging and feature randomness to shape an uncorrelated forest of decision trees. This randomness creates a subset of features which guarantees low correlation among decision trees [82]. Figure 2 portrays how RF operates.

The fact that each decision tree operates on a different subset of data allows the algorithm to handle nonlinear datasets, missing or “noisy” clinical data better than SVM. Moreover, it provides clinically interpretable feature importance for stratification.

Nevertheless, RF is less precise on high-dimensional data in comparison to SVM [83]. Another disadvantage of RF is that it is impossible to replicate predictions without an actual forest. Thus, future predictions require the original forest with the original data or a new forest that mimics the predictions with synthetic data. Model development is also more complex as each data set would generate a different model, and there is no known way to compare model parameters. This leads to difficulty in the validation of prediction models in separate population cohorts. Another impediment of RF is that there exists a possibility for models with entirely different component variables to result in nearly identical predictions. What is more, it is possible for models with identical component variables to result in distinct predictions owing to differences in construction, such as variable choice [84].

Numerous studies have proven the practical utility of SVM models in cardiology. Hermans et al. applied an SVM-based model with T-wave morphologic analysis for long QT syndrome diagnosis with sensitivity and specificity of 82.0% and 86.1%, outperforming traditional Qtc-based methods [85]. Similarly, Arsanjani et al. used an SVM model in the detection of coronary artery disease with myocardial perfusion SPECT imaging, highlighting much improved diagnostic accuracy compared to conventional perfusion metrics [86]. Random forest algorithms have demonstrated high efficacy in HF diagnostics. In a study by Wang et al., a Random Forest model predicting coronary artery disease achieved an AUC of 0.948, with a sensitivity of 90.0% and a specificity of 95.4% [87].

Yuan et al. invented an RF-based model for HF prediction, including biomarkers such as BNP, CK-MB, sST2, and Gal-3. The model demonstrated the utility of RF in integrating biomarker datasets, with sensitivity and specificity of 91.5% and 96.7%, respectively [88].

### 5.2. Utilization of Unsupervised Learning for Discovery of New Subtypes

The other category of ML, unsupervised learning, relies on algorithms for analysis and clusters of unlabeled data sets. This model demonstrates the ability to identify similarities and differences, hidden patterns, or data groupings without the necessity of human intervention [89]. Clustering, dimensionality reduction, and association are the main domains of unsupervised learning approaches.

Within this branch of ML, clustering using K-means exhibits the ability to classify and organize complex data into groups according to their patterns or similarities. The k-means clustering algorithm divides components into groups based on their similarity. The letter k indicates the quantity of groups, e.g., if k equals 6, it means that there are six groups. The unlabeled datasets are divided into unique clusters with comparable qualities for each data point. Difficulty may be posed by the location of K centers for each set of data, known as cluster centroids. After presentation of a new data point, the algorithm will employ metrics in order to determine the cluster the data point pertains to [86]. This algorithm allows the detection of underlying trends and patterns. Moreover, it enables recognition of new variants or subtypes within complex heterogeneous groups. In the event of excessive sophistication of datasets, dimensionality reduction can be applied. This algorithm demonstrates that extensive datasets may be represented by fewer features while capturing the core characteristics [90]. However, the k value (number of groups in a network) and various other original inputs are greatly impacted by the outcome. Moreover, the final result is significantly influenced by the order in which the data is input. The algorithm’s susceptibility to changes by scaling given data, implementing normalization, or standards also has a great impact on the output [86].

Clustering’s ability to recognize data-driven subgroups that share underlying features may open new pathways in cardiology. The algorithm can detect new biological markers and cellular subpopulations which may be linked to disease. By identifying these new variants, we can have more specific treatment options directed towards particular subtypes in these conditions [91], allowing patient selection for trials by targeting biologically heterogeneous groups and earlier detection of heart dysfunctions or arrhythmia-related deterioration. Shah et al. applied hierarchical clustering to patients with heart failure, distinguishing phenogroups with different clinical courses, which significantly differed in mortality risk and hospitalization time [92]. ML-based clustering of atrial fibrillation populations isolated clinically distinct patient clusters with different responses to rhythm-control therapies, with different outcomes, which highlighted the potential of unsupervised learning in personalized medicine [93].

### 5.3. DL in Omics Integration

The biological interactions described in Section 2, particularly the multi-layered regulatory networks of miRNAs, lncRNAs, and exosomal signaling, are fundamentally non-linear and highly complex. Consequently, traditional linear statistical models often fail to capture these intricate patterns, necessitating the application of DL algorithms. DL architectures, through their multi-layered neural networks, are uniquely capable of mapping these sophisticated molecular dependencies and extracting meaningful biological signals from the inherent noise of multi-omics data.

DL, a sophisticated subset of ML, utilizes NN designed to resemble the human brain’s architecture by composing layers of ‘neurons’ that perform sequential mathematical operations [94]. Multiplying and stacking these layers allows automatic abstractions. Each layer abstracts its inputs, providing the following layer with a data representation that is easier to work with in the context of the task being solved [95]. NNs have the ability to integrate multi-omics data as the algorithm is enabled to learn nonlinear relationships and latent biological factors that non-AI methods miss. This DL model allows analysis of vast datasets while avoiding multi-modal overfitting. However, to ensure clinical utility, these multi-omics pipelines must first rigorously address technical and biological variability. Pre-processing steps should include batch-effect correction and the adjustment for confounders—such as age, sex, and medication (e.g., statins)—which can significantly skew metabolic and transcriptomic profiles [96].

Beyond pre-processing, the choice of integration strategy is critical. Current research is increasingly moving toward sophisticated frameworks like Multi-Omics Factor Analysis (MOFA) and DIABLO, which allow for the simultaneous identification of correlations across disparate data layers [97]. Furthermore, late-fusion ML approaches are being employed to integrate high-level features from independent omics models, providing more robust predictions for specific CVD phenotypes, such as HF or atherosclerotic plaque stability, compared to early-fusion methods [98].

In contrast to these technical strategies, there is the ongoing challenge of model interpretability. The internal workings of NN are unknown, as only their input and output are specified. This phenomenon is referred to as ‘black-box’. Its occurrence generates skepticism among medical specialists regarding the accuracy or explainability of the ML technique and doubts whether outputs of NN are correct [99]. The emergence of uncertainty led to the creation of a new domain of AI known as XAI. The main aim of XAI is to enable people to understand model predictions [100]. It uses various explainability methods that can be divided into multiple categories. One of them is Locally Interpretable Model–Agnostic Explainer (LIME). This method is focused on explaining individual predictions of black-box models by investigating changes in outputs when diverse data are placed into an ML model, which leads to the creation of a new dataset with different samples and black-box predictions, enabling the emergence of a new interpretable model [101]. The underlying concept is to predict the behavior of a model by using a smaller, more understandable model called an interpretable model. LIME’s versatility and adaptability enable it to generate explanations for a wide range of ML models, including DL NN [102].

In contrast to LIME, there also exists another XAI algorithm known as SHAP. It utilizes game theory within ML models for interpretability. The respective characteristic has a Shapley value assigned, which acts as an importance value based on the fair share of contributions made by the feature. SHAP is theoretically grounded and consistent, and used for structured or tabular data explanations mostly [103]. However, it is limited in establishing causal relationships, which poses an issue in time-series data where confounding and sparsity complicate interpretability, such as in applications in various medical fields. What is more, the SHAP values only relate to the predictive influence of exposures [104].

Kwon et al. invented a deep neural network model, which integrates electrocardiographic and clinical data for HF detection. The study underscored the ability of DL architectures to extract latent nonlinear patients, which cannot be detected by traditional statistical approaches [105]. Hannun et al. applied a deep neural network to ECG analysis for arrhythmia classification, obtaining high performance with an average AUC exceeding 0.97. The study demonstrated the capability of DL models to process large-scale electrophysiological data with high precision [106]. In cardiovascular medicine, XAI models have been used to identify biomarkers, ECG abnormalities, and imaging characteristics driving model predictions, facilitating clinical translation of AI [107].

### 5.4. AI in Detecting CVDs

AI and ML technologies are more commonly implemented in the studies of CVDs, especially in molecular data analysis. Modern technologies such as Proteomics, Genomics, and Transcriptomics have enabled the gathering of a significant amount of data, which requires advanced, easy-to-use data analysis techniques. One of the examples is a Random Forest RNA seq model, enabling the identification of typical genes correlated with advanced HF progression, and clustering the patients into high and low risk of progression, based on the gene expression profiles. Moreover, a deep neuronal network (DNN) is able to analyze transcriptomic data, leading to the detection of molecular signs of HF with increased prediction precision, compared to other methods [108]. An integrated approach was proposed by Liu et al., who implemented ML to conjunct transcriptomic and proteomic data in heart arrhythmias, distinguishing atrial fibrillation and normal sinus rhythms [109]. Poligenic risk models based on AI use hundreds of genetic variants from the GWAS database for the purpose of stratification of individual risk of suffering from CAD in the future [110]. Another tool has been introduced by Wang et al., using Random Forests for the detection of gene expression in the blood serum, participating in the pathogenesis of CAD, proposing a possible future diagnostic tool [87]. GWAS and expression Quantitative Trait Loci (eQTL) databases integration using ML enabled highlighting genes acting as key players in the pathogenesis of CVDs [111]. AI and ML data analysis techniques are a key addition to the overall clinical data analysis arsenal. Due to the overwhelming amount of data analyzed by a single physician, AI and ML techniques provide support in everyday clinical practice and remain a vital contributor to the global introduction of the individual approach in CVD treatment. Gene databases such as Gene Expression Omnibus (GEO) can serve as a source of potential genes contributing to the development of CVDs, being potential diagnostic targets. Detecting concrete pathogenic genes tailors the way to individualized treatment based on the presence of low or high-risk genes [112].

**Table 2:** Classification and clinical application of machine learning techniques in cardiovascular multi-omics.

ML Category	Algorithms Mentioned	Function and Mechanism	Clinical Applications and Outcomes	References
Supervised Learning	SVM	Uses labeled datasets as “ground truth” to learn feature-label correspondences; updates parameters to classify and predict outcomes.	Stratification of HF risk; identification of genes participating in CAD pathogenesis; distinguishing AF from sinus rhythm.	[76,81,83,84,85,86]
	Random Forest			
	KNN			
	Decision Trees			
	Logistic Regression			
Unsupervised Learning	Clustering	Analyzes unlabeled data to identify hidden patterns, similarities, or data groupings without human intervention.	Detection of new cardiovascular subtypes, cellular subpopulations, or heart dysfunction/arrhythmia deterioration; patient selection for trials.	[86,89,90,91,92,93]
	Dimensionality Reduction			
Deep Learning	Neural Networks	Uses multi-layered networks to map non-linear molecular dependencies and extract signals from complex multi-omics noise.	Analysis of transcriptomic data to detect molecular signs of HF with high precision; integration of multi-modal omics data.	[94,96,108]
	Deep Neural Networks			
Explainable AI	LIME	Investigates changes in outputs when diverse data are input to make “black-box” predictions understandable to people.	Providing explainability for DL models to address skepticism among medical specialists regarding the accuracy of AI outputs.	[101,102,103]
Integrative AI Approaches	Polygenic Risk Models	Combines data from various sources (GWAS, eQTL, GEO, Proteomics, Transcriptomics).	Individual risk stratification for CAD; identifying genes correlated with advanced HF progression; global introduction of individualized treatment.	[87,110,111,112]
	RF RNA-seq Models	Uses gene expression profiles to identify typical genes and classify patient risk.	Identifying genes correlated with advanced HF progression; high/low risk clustering.	[81,82,83,84,88]

AF—Atrial Fibrillation; AI—Artificial Intelligence; CAD—Coronary Artery Disease; DL—Deep Learning; eQTL—expression Quantitative Trait Loci; GEO—Gene Expression Omnibus; GWAS—Genome-Wide Association Studies; HF—Heart Failure; KNN—k-Nearest Neighbor; LIME—Locally Interpretable Model–Agnostic Explainer; RF RNA-seq Models—Random Forest RNA-sequencing Models; SVMs—Support Vector Machines.

## 6. Multi-Omics Biomarkers and AI in Modern Cardiology: Towards Fully Personalized Medicine

### 6.1. The Growing Importance of Multi-Omics Biomarkers in Modern Cardiology

Over the past decade, cardiovascular research has undergone a significant transformation thanks to the development of multi-omics technologies, such as genomics, epigenomics, transcriptomics, proteomics, and metabolomics. These platforms enable a comprehensive, multilayered understanding of CVD biology, going beyond traditional approaches based on single biomarkers or clinical indicators. Integrating data from different molecular layers allows researchers to capture complex cellular and molecular interactions that underlie the onset, progression, and clinical heterogeneity of CVD [113].

Genomics provides information on inherited genetic variation and disease susceptibility, while epigenomics reveals how environmental factors and lifestyle modulate gene activity through DNA methylation, histone modifications, and changes in chromatin accessibility [113]. Epigenomics, which studies heritable changes in gene regulation without altering the DNA sequence, can greatly benefit from AI and ML approaches. These methods allow detection of complex patterns in DNA methylation, histone modifications, and chromatin accessibility, identifying disease-associated epigenetic signatures that may be missed by traditional analyses. Integrating AI with epigenomic data thus enhances the identification of novel biomarkers and predictive models for CVDs, supporting personalized medicine approaches [113]. Transcriptomics captures dynamic changes in gene expression, including non-coding regulators such as microRNAs, which control pathways related to inflammation, myocardial remodeling, and metabolism. Proteomics translates these signals into functional proteins that influence cardiac contractility, oxidative stress response, extracellular matrix remodeling, and immune system activity. Metabolomics, in turn, measures small molecules and metabolic intermediates, reflecting the current physiological state, energy metabolism, and systemic homeostasis, which allows detection of subtle changes in response to ischemia, hypertrophy, or inflammatory states [61,113,114].

Integrating these layers enables the reconstruction of the molecular networks of CVD, identifying how genetic predispositions interact with transcriptomic regulation, how these signals are manifested at the protein level, and how metabolomic changes affect cellular function. This approach reveals interactions and regulatory networks that remain invisible in single-omics studies. Multi-omics analyses can identify key molecular pathways, epigenetic markers, and metabolic signatures predictive of adverse clinical outcomes [113,114].

Despite the immense complexity of the data, modern computational biology methods and AI, including ML and DNN, allow the extraction of actionable biological insights and the discovery of new biomarkers. This approach supports mechanistic understanding of CVD, identification of potential therapeutic targets, and the development of personalized medicine tailored to the unique molecular profile of each patient [113,114].

Circulating miRNAs, as part of the transcriptomic layer, are an example of non-coding regulators that modulate gene expression, controlling fibrosis, apoptosis, inflammation, and cardiac remodeling—processes that are central to the pathogenesis of many CVDs. Proteomic biomarkers such as NT-proBNP, ST2, and GDF-15 remain fundamental in evaluating myocardial wall stress, oxidative injury, and post-inflammatory remodeling [87].

NT-proBNP is routinely used to assess the severity of HF, while markers such as ST2 and GDF-15 reflect myocardial strain and the activity of inflammatory signaling pathways [111]. Metabolomic profiling enables detection of metabolic disturbances associated with HF, including levels of TMAO, acylcarnitines, branched-chain amino acids, and other metabolites linked to mitochondrial dysfunction and energy dysregulation [112]. Analysis of blood metabolites allows not only monitoring of the patient’s current physiological state but also the detection of subtle metabolic changes that may indicate disease progression or response to therapy [111,112].

Studies have shown that higher levels of SDMA, putrescine, methionine sulfoxide, and 5-hydroxylysine are associated with worse cardiovascular outcomes, including increased risk of hospitalization and cardiovascular mortality [112]. Conversely, higher levels of amino acids such as tryptophan and histidine exhibit protective effects, suggesting their potential role in modulating inflammatory responses, oxidative stress, and cardiac remodeling [113]. Furthermore, these metabolomic signatures may reflect both energy disturbances in cardiomyocytes and endothelial dysfunction or alterations in the gut microbiome, highlighting the complexity of pathophysiological processes in HF [111,112].

Integrating metabolomic data with other omics layers, including proteomics and transcriptomics, allows not only more precise risk prediction but also the identification of novel biomarkers and potential therapeutic targets [111,112]. This approach enables the development of predictive models that account for nonlinear interactions and complex regulatory networks, supporting personalized treatment strategies for patients with HF [111,112].

These findings underscore the importance of metabolomic profiling in risk stratification and personalized treatment strategies. Integrating these heterogeneous datasets generates substantial analytical complexity, which can only be effectively addressed through AI and ML frameworks capable of handling high-dimensional data. Such integration enables both biomarker discovery and the development of predictive models that account for nonlinear interactions and multi-layered regulatory networks [110].

### 6.2. AI-Driven Integration of Multi-Omics Data and Biomarkers

AI plays a pivotal role in analyzing high-dimensional multi-omics datasets, enabling the identification of novel disease signatures, the construction of robust risk-prediction models, and the elucidation of complex cross-omics biological relationships [113]. By applying ML algorithms to large-scale datasets, researchers can detect subtle, nonlinear patterns that often remain hidden from traditional statistical methods. These algorithms, which include random forests, support vector machines, and neural networks, are particularly effective in handling the complexity of multi-omics data, integrating diverse molecular layers to produce more accurate and individualized risk assessments [115].

For instance, predictive models that combine NT-proBNP with miRNA signatures have been shown to substantially improve the forecasting of heart-failure-related hospitalizations and mortality [115]. Similarly, the study by Sun et al. (2025) [116] demonstrated the application of multiple ML algorithms to predict worsening HF in chronic HF patients, using high-dimensional clinical and molecular features. Their approach, enhanced by interpretable methods, allowed not only the identification of key risk factors but also the development of a clinically applicable risk-scoring tool, illustrating how ML can bridge the gap between complex molecular data and practical clinical decision-making [116].

Hybrid multi-omics models, which integrate proteomic, transcriptomic, and metabolomic information, outperform single-marker approaches in predicting adverse post-myocardial-infarction remodeling and progressive left ventricular dysfunction [75]. Deep-learning architectures tailored to RNA sequencing or high-dimensional proteomic data further enable the interpretation of entire regulatory networks, rather than focusing on isolated biomarkers, providing a systems-level view of disease mechanisms and enhancing the precision of prognostic models [115].

**Figure 3:**
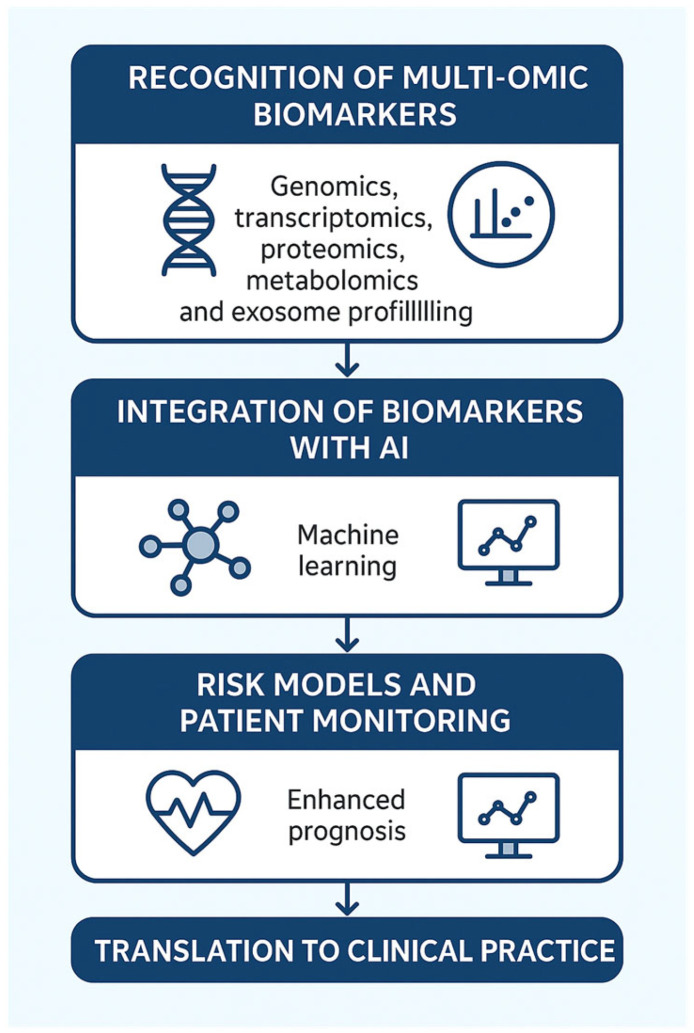
The figure presents the process of integrating multi-omic biomarkers with artificial intelligence (AI) algorithms for clinical risk prediction, prognosis assessment, and patient monitoring. Legend: Multi-omic data, including genomics, transcriptomics, proteomics, metabolomics, and exosome profiling, are integrated using machine learning algorithms. AI-based models analyze interactions between biomarkers and identify signatures associated with disease progression and clinical outcomes. The resulting predictive models support risk stratification, prognosis evaluation, and the implementation of precision medicine in clinical practice.

The integration of multi-omics data plays a key role in drug discovery and the identification of novel therapeutic targets in HF. By combining information from genomics, proteomics, transcriptomics, metabolomics, and epigenomics, researchers can identify molecular pathways critical for disease progression and pinpoint potential therapeutic targets. This approach also enables the discovery of predictive biomarkers for drug response and adverse effects, supporting personalized treatment strategies tailored to a patient’s molecular profile (Figure 3). Furthermore, multi-omics data can inform all stages of drug development—from preclinical testing to clinical trial design—accelerating the creation of more effective therapies [114].

### 6.3. Clinical Translation—Risk Models and Post-MI Monitoring

Advanced risk-prediction models that integrate NT-proBNP with fibrosis- and stress-related miRNAs improve prognostic precision over conventional biomarkers alone [114]. Circulating miRNAs are robust predictors of extracellular matrix remodeling, inflammation, and apoptosis, and their inclusion in predictive models substantially improves patient stratification [117].

AI-driven multi-omics analyses can forecast adverse remodeling, identify patients requiring intensified clinical surveillance, and predict functional recovery trajectories post-MI [118]. Machine-learning tools now support assessment of tissue regeneration, detection of subtle metabolic alterations, and prediction of therapeutic response, representing a major step toward fully personalized cardiac care [113].

Furthermore, dynamic multi-omics monitoring can optimize therapy by tracking molecular and metabolic changes in real time, improving clinical decision-making and patient outcomes [119].

### 6.4. Translational Challenges—Standardization, Data Quality, and Regulatory Considerations

Despite the potential of multi-omics biomarkers, several challenges limit their clinical translation. Lack of standardized protocols, especially for miRNA extraction, high-throughput proteomics, and advanced metabolomics, results in inconsistencies between laboratories [117]. Variability between analytical platforms and bioinformatic pipelines further complicates reproducibility [113].

Moreover, the high costs associated with generating and analyzing multi-omics data, including DNA/RNA sequencing, proteomics, and metabolomics, as well as the requirement for advanced computational infrastructure, significantly limit the availability of these biomarkers in routine clinical practice. As a result, their accessibility is much lower compared to widely used and inexpensive biomarkers such as cardiac troponins [116,120,121,122,123].

In addition, AI-based tools applied in cardiovascular multi-omics analyses have several limitations. ML models can exhibit bias if trained predominantly on data from a single population, such as Caucasian patients, resulting in reduced accuracy for other ethnic groups. Other challenges include the lack of interpretability of complex models (“black box”), potential amplification of health disparities, and the need for rigorous validation across diverse populations [120,121,122,123].

Regulatory agencies such as the FDA and EMA require AI-based tools to demonstrate transparency, reproducibility, bias mitigation, and population-level validation. Ethical and privacy concerns regarding genomic and metabolomic datasets necessitate robust governance mechanisms to protect patient data while enabling clinical innovation [119].

### 6.5. The Future—Digital Biomarker Panels and Fully Personalized Cardiovascular Medicine

Emerging evidence suggests that the future of cardiology lies in digital biomarker panels that integrate multi-omics data with imaging modalities, clinical parameters, and physiological data from wearable devices [116]. AI systems capable of dynamically analyzing these integrated datasets could detect early signs of HF decompensation, track disease trajectories, and guide individualized therapeutic interventions [120].

miRNAs are not only biomarkers but also potential therapeutic targets. Integrating miRNA, proteomics, and metabolomics into digital biomarker panels enables proactive, fully personalized cardiovascular care, in line with precision medicine principles [122]. Continuous monitoring and AI-driven analysis of multidimensional biological profiles may optimize treatment strategies and improve patient outcomes. As cardiology moves toward digital biomarker panels and precision strategies, the distinction between current reactive models and the proposed proactive multi-omics approach becomes clear (see Table 3).

## 7. Conclusions

The integration of multi-omics with AI represents a significant shift in cardiovascular medicine, moving toward a granular, mechanistic understanding of disease. By synthesizing data from genomics to metabolomics, researchers are bridging the diagnostic gap left by conventional biomarkers, which often fail to detect subclinical changes.

AI is central to this evolution, navigating high-dimensional data to identify subtle signatures—such as exosomal miRNA or metabolic shifts—that precede clinical symptoms. To accelerate the clinical translation of these technologies, several critical steps must be taken.

First, institutions must establish unified protocols for the collection and storage of “omics” samples to ensure data reproducibility across global clinical sites, particularly for volatile metabolites and miRNAs. Furthermore, researchers should prioritize the inclusion of non-Caucasian cohorts in training datasets to eliminate algorithmic bias and ensure that AI-driven risk models remain accurate across all ethnicities.

Clinicians should also move toward “multi-marker panels” that combine gold-standard proteins, such as NT-proBNP, with mechanistically relevant ncRNAs, rather than searching for a single standalone biomarker. Finally, developers must implement XAI frameworks to transition from “black-box” models to transparent systems, allowing physicians to understand the biological rationale behind an AI’s risk prediction.

**Figure 4:**
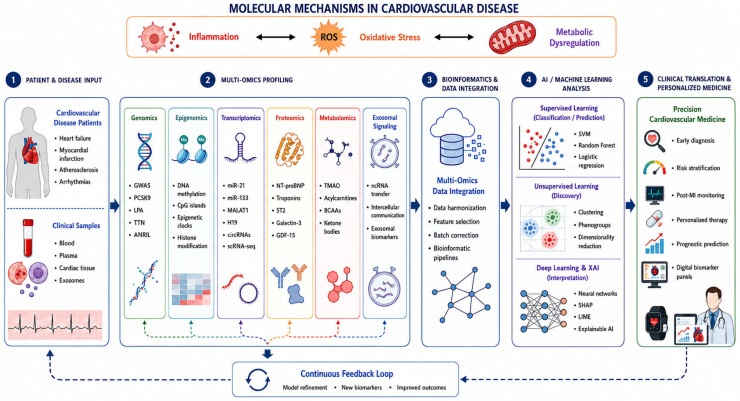
Integrated workflow of multi-omics and artificial intelligence approaches in cardiovascular disease research.

While the synergy of multi-omics and AI holds potential for proactive care, its success depends on these interdisciplinary efforts to establish rigorous standards and transparency before routine clinical adoption. Figure 4 represents the integrated workflow of multi-omics and AI approaches in CVD research.

## Figures and Tables

**Table 1:** Summary of biomarkers in circulation.

Domain	Biomarkers	Associations	References
Genomics	*PCSK9*	LDL receptor regulation; hypercholesterolemia risk	[47]
	9p21.3 (*ANRIL*)	CAD/MI risk locus	[46]
	*LPA*	Elevated Lp(a), MI, and stroke risk	[48]
	*TTN*	Dilated cardiomyopathy, arrhythmias	[49]
	*LMNA*	Conduction disorders, cardiomyopathy	[50]
Transcriptomics	*miR-21*	Fibrosis, inflammation, HF progression	[54]
	*miR-1*/*miR-133a*/*miR-208*	Myocardial injury, infarction markers	[55]
	*miR-499*	Cardiomyocyte stress, acute MI	[55]
	MALAT1	Cardiomyocyte apoptosis	[56]
Proteomics	BNP/NT-proBNP	HF diagnosis and prognosis	[61]
	hs-troponins	Gold-standard cardiac injury markers	[60]
	Galectin-3	Fibrosis and remodeling	[62]
	GDF-15	Stress cytokine, prognostic value in HF	[63]
	ST2(sST2)	Remodeling and risk stratification	[59]
	C-reactive protein (CRP)	Inflammation, CAD risk	[59]
Metabolomics	Acylcarnitines	Mitochondrial dysfunction, HF	[23]
	TMAO	Gut-heart axis, CAD risk	[65]
	Branched-chain amino acids	Insulin resistance, cardiometabolic risk	[66]
	Ketone bodies	Potential HF severity marker	[67]
	Ceramides	Risk stratification for cardiovascular events	[64]
Epigenomics	CpG methylation	Regulation of inflammation genes	[70]
	Histone modifications	Chromatin remodeling, cardiovascular aging	[68]
	Epigenetic clocks	Biological aging, mortality prediction	[69]
Immunomics/inflammatory markers	IL-6	Inflammation, CAD progression	[59]
	TNF-α	Cardiomyocyte apoptosis, remodeling	[59]
Microbiome/Multiomics interactions	Gut microbial metabolites	TMAO, SCFas influence on CAD/HF	[65]
	Microbiome signatures	Personalized cardiovascular risk	[65]

BNP—B-type natriuretic peptide; CAD—coronary artery disease; CRP—C-reactive protein; GDF-15—growth differentiation factor 15; HF—heart failure; hs-troponins—high-sensitivity cardiac troponins; IL-6—interleukin 6; LDL—low-density lipoprotein; LMNA—lamin A/C; Lp(a)—lipoprotein(a); MI—myocardial infarction; miR—microRNA; NT-proBNP—N-terminal pro–B-type natriuretic peptide; PCSK9—proprotein convertase subtilisin/kexin type 9; sST2—soluble suppression of tumorigenicity 2; TMAO—trimethylamine N-oxide; TNF-α—tumor necrosis factor alpha; TTN—titin gene.

**Table 3:** Conventional vs. precision cardiovascular medicine.

Feature	Conventional Clinical Framework	Emerging Multi-Omics and AI Framework
Risk Detection Stage	Primarily reactive; markers often become abnormal only after significant disease progression [3].	Proactive; identifies subclinical and mechanistically relevant changes at early stages [2,5].
Biomarker Specificity	Restricted; LDL-C and hsCRP can be influenced by non-cardiac conditions or lack a genetic context [3].	High; utilizes cell-specific signatures such as miRNAs, lncRNAs, and specific lipid species [24,27,28,29,30,31,32,33].
Genetic Insight	Limited to traditional risk factors or rare single-gene disorders [46].	Comprehensive; integrates SNPs (*LPA*, *PCSK9*, *APOE*) and structural variants (*TTN*, *LMNA*) to stratify risk [44,45,47].
Metabolic Assessment	Static measures of systemic lipids and glucose [3].	Dynamic profiling of mitochondrial function (acylcarnitines) and gut microbiome (TMAO) [23,62].
Data Interpretation	Reliance on conventional diagnostic tools and linear statistical models [2,3].	Integration of high-dimensional “omics” data through AI/ML (RF, SVM, DNN) [4,82,112].
Management Goal	Standardized post-diagnosis medical care and population-level strategies [1,2].	Truly personalized preventative strategies and precision cardiovascular medicine [5,116,118].

AI—Artificial Intelligence; APOE—Apolipoprotein E gene; DNN—Deep Neural Network; hsCRP—High-Sensitivity C-Reactive Protein; LDL-C—Low-Density Lipoprotein Cholesterol; LMNA—Lamin A/C gene; lncRNAs—Long non-coding RNAs; LPA—Lipoprotein(a) gene; miRNAs—MicroRNAs; ML—Machine Learning; PCSK9—Proprotein Convertase Subtilisin/Kexin type 9; RF—Random Forest; SNPs—Single Nucleotide Polymorphisms; SVMs—Support Vector Machines; TMAO—Trimethylamine N-oxide; TTN—Titin gene.

## Data Availability

No new data were created or analyzed in this study. Data sharing is not applicable to this article.

## Abbreviations

The following abbreviations are used in this manuscript:

AI	Artificial Intelligence
*ALOX5AP*	Arachidonate 5-Lipoxygenase-Activating Protein
AMI	Acute Myocardial Infarction
*APOE*	Apolipoprotein E
ASCVD	Atherosclerotic Cardiovascular Disease
BCAAs	Branched-Chain Amino Acids
CAD	Coronary Artery Disease
CHD	Coronary Heart Disease
circRNAs	Circular RNAs
CVD/CVDs	Cardiovascular Diseases
DCM	Dilated Cardiomyopathy
DNN	Deep Neuronal Network
DL	Deep Learning
eQTL	Expression Quantitative Trait Loci
GBD	Global Burden of Disease
GDF-15	Growth Differentiation Factor-15
GEO	Gene Expression Omnibus
GWAS	Genome-Wide Association Studies
HF	Heart Failure
HNF1	Hepatocyte Nuclear Factor 1
hsCRP	High-Sensitivity C-Reactive Protein
IKKβ	Inhibitor of Nuclear Factor Kappa-B Kinase Subunit Beta
IL-1β	Interleukin-1β
JNK	Jun N-Terminal Kinase
KNN	K-Nearest Neighbor
LDL-C	Low-Density Lipoprotein Cholesterol
LIME	Locally Interpretable Model–Agnostic Explainer
LMNA	Lamin A/C gene
lncRNAs	Long Noncoding RNAs
Lp(a)	Lipoprotein(a)
MAPK	Mitogen-Activated Protein Kinase
MI	Myocardial Infarction
ML	Machine Learning
miRNAs	microRNAs
MOFA	Multi-Omics Factor Analysis
mRNA	Messenger RNA
NADPH	Nicotinamide Adenine Dinucleotide Phosphate Oxidases
ncRNA	Non-coding RNA
NF-κB	Nuclear Factor Kappa-Light-Chain-Enhancer of Activated B Cells
NNs	Neural Networks
NO	Nitric Oxide
NOX	NADPH oxidase
PCSK9	Proprotein Convertase Subtilisin/Kexin type 9
PGC-1α	Peroxisome Proliferator-Activated Receptor Gamma Coactivator 1-Alpha
pri-miRNA	Primary miRNA
RF	Random Forest
ROS	Reactive Oxygen Species
SCD	Sudden Cardiac Death
scRNA-seq	Single-Cell RNA Sequencing
SDI	Socio-Demographic Index
siRNA	Interfering RNA
SIRT1	Sirtuin 1
SNPs	Single Nucleotide Polymorphisms
SOM	Self-organizing map
SREBP	Sterol Regulatory Element-Binding Protein
SVMs	Support Vector Machines
TMAO	Trimethylamine N-oxide
TNF-α	Tumor Necrosis Factor α
TTN	Titin Gene
3′-UTRs	3′ Untranslated Regions
VSMCs	Vascular Smooth Muscle Cells
XAI	Explainable Artificial Intelligence
